# *Aedes albopictus* gut symbiotic bacterium *Bacillus cereus* improves its deltamethrin resistance

**DOI:** 10.1186/s13071-025-07229-5

**Published:** 2026-01-09

**Authors:** Yingbo Sun, Qingyun Huang, Yunfei Zhou, Guofa Zhou, Jiabao Xu, Saifeng Zhong, Tianya He, Yu Jiang, Suhua Liu, Daibin Zhong, Gang Lu, Tingting Li, Yiji Li

**Affiliations:** 1 Key Laboratory of Tropical Translational Medicine of Ministry of Education, College of Basic Medical Sciences, Hainan Medical University, Haikou, 571199 China; 2 Department of Pathogen Biology, College of Basic Medical Sciences, Hainan Medical University, Haikou, 571199 China; 3 Hainan Medical University-The University of Hong Kong Joint Laboratory of Tropical Infectious Diseases, Hainan Medical University, Haikou, 571199 China; 4 Program in Public Health, College of Health Sciences, University of California at Irvine, Irvine, CA 92617 USA; 5 Department of Immunology and Microbiology, School of Basic Medical Sciences, Zhejiang Chinese Medical University, Hangzhou, 310053 Zhejiang China

**Keywords:** *Aedes albopictus*, Gut microbes, *Bacillus cereus*_HL4.2, Deltamethrin resistance

## Abstract

**Background:**

*Aedes albopictus* is a highly invasive vector for a variety of pathogens. The intensive use of insecticides has led to the widespread insecticide resistance in *Ae. albopictus* populations worldwide, compromising disease vector control efforts. We investigated whether the mosquito gut symbiotic bacterium *Bacillus cereus* reduces deltamethrin susceptibility in *Ae. albopictus* and elucidated the underlying mechanisms.

**Methods:**

World Health Organization (WHO) standard tube bioassays were conducted to assess deltamethrin resistance status in both laboratory and field *Ae. albopictus* populations before and after oral infection with *Bacillus cereus*_HL4.2 (*B. cereus*_HL4.2). We measured enzymatic activities of three major detoxification enzyme families (cytochrome P450 monooxygenases, glutathione *S*-transferases [GSTs], and carboxylesterases) as metabolic markers. Transcriptomic profiling via RNA sequencing (RNA-seq) identified genes differentially expressed upon *B. cereus* infection, with subsequent validation by quantitative reverse-transcription PCR. In vitro assays assessed the direct deltamethrin-degrading capacity of *B. cereus_*HL4.2, and green fluorescent protein (GFP)-labeled bacterial strains tracked bacterial persistence and transmission through mosquito developmental stages.

**Results:**

Oral infection with *B. cereus*_HL4.2 significantly increased the survival rate of laboratory-susceptible *Ae. albopictus* after deltamethrin exposure (from 7.6 ± 2.0% to 31.3 ± 4.3%) upon lethal insecticide exposure. *B. cereus*_HL4.2 infection elevated detoxification enzyme activities: cytochrome P450s increased 1.39-fold and GSTs increased 1.21-fold. Transcriptomic analysis revealed upregulation of genes related to the cAMP signaling pathway and purine metabolism following *B. cereus*_HL4.2 infection, while genes associated with ABC transporter and sensory signaling pathways were primarily downregulated. In vitro studies demonstrated that *B. cereus*_HL4.2 possesses direct deltamethrin-degrading capacity. GFP-tracking confirmed that *B. cereus_*HL4.2 colonizes the mosquito gut during larval development and persists through adult emergence.

**Conclusions:**

*Bacillus cereus*_HL4.2 infection reduces deltamethrin susceptibility in *Ae. albopictus* primarily through two complementary mechanisms: (*i*) metabolic upregulation of detoxification enzymes and related genes, and (*ii*) direct enzymatic degradation of deltamethrin. Genetically modifying *B. cereus*_HL4.2 may offer a potential strategy for managing insecticide resistance in mosquitoes.

**Graphical Abstract:**

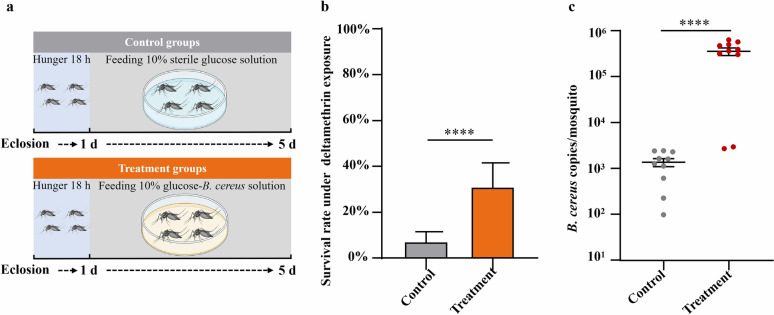

**Supplementary Information:**

The online version contains supplementary material available at 10.1186/s13071-025-07229-5.

## Background

Each year, tens of millions of people are infected with arthropod-borne viruses [1–3]. The *Aedes albopictus* (Skuse, 1894) mosquito is an essential vector of dengue viruses (DENV), chikungunya virus (CHIKV), Zika virus (ZIKV), and other medically important arboviruses [4–6]. Originating from the tropical rainforests of southeast Asia, *Ae. albopictus* has undergone global range expansion in the past few decades, and is now established on five continents [7, 8]. Currently, vaccines are unavailable or ineffective for most *Aedes*-borne viruses, including CHIKV and ZIKV [9]. Consequently, vector control through insecticidal management remains the primary disease prevention strategy. Pyrethroids are favored owing to their selective mammalian safety and demonstrated effectiveness against mosquitoes [10]. However, mosquitoes have developed varying degrees of insecticide resistance as a consequence of prolonged exposure globally [11–13]. This resistance phenomenon presents an increasingly challenge for mosquito vector control. The principal mechanisms of insecticide resistance in mosquitoes include behavioral avoidance of insecticide exposure, increased cuticle thickness limiting insecticide penetration, target-site mutations, and metabolic resistance through upregulation of detoxification enzyme systems (cytochrome P450s, glutathione *S*-transferases, and carboxylesterases) [14–16]. Emerging evidence reveals that mosquito gut microbiota may play a role in modulating insecticide resistance phenotypes [17], representing a novel and underexplored dimension of the insecticide resistance problem.

The insect gut microbiota plays crucial physiological roles in host growth, development, and immunity [18–20]. Mounting evidence demonstrates a mechanistic link between alterations in insect gut microbial composition and enhanced insecticide resistance [21–23]. Gut bacteria may confer resistance advantages to their hosts through two primary mechanisms: degrading insecticidal compounds and/or altering the expression of host genes [23, 24]. Despite these findings, knowledge about the structure and functional contributions of mosquito gut microbiota to insecticide resistance and pathogen transmission remains incomplete [22, 25]. Most prior studies used laboratory-reared susceptible or insecticide-selected mosquitoes to study microbiota–insecticide resistance interactions, limiting their generalizability to field populations. Field mosquitoes likely harbor markedly different microbiota assemblages compared with standardized laboratory strains owing to environmental heterogeneity and dietary variation [22]. Moreover, the molecular and biochemical mechanisms underlying gut-microbiota-mediated insecticide resistance in mosquitoes remain poorly understood, highlighting a significant knowledge gap that warrants further investigation.

Understanding the mechanisms by which mosquito gut microbiota modulate insecticide resistance may provide novel strategies for mosquito control and disease prevention. In our previous study, *16S* rDNA sequencing revealed significantly different gut microbial diversity in deltamethrin-resistant field larvae compared with susceptible larvae. This analysis identified 25 distinct bacterial species from resistant larvae mosquito populations [26]. Among them, we identified a symbiotic strain, *Bacillus cereus*_HL4.2, which conferred reduced susceptibility to deltamethrin, thereby significantly increasing mosquito survival. In the present study, we assessed the role of *B. cereus*_HL4.2 in reducing deltamethrin susceptibility in *Ae. albopictus* and explored the potential mechanisms (metabolic detoxification genes regulation and/or direct degradation of pesticides) underlying this resistance through multiple complementary approaches. Our findings broaden understanding of the regulatory role of mosquito gut symbiotic bacteria in insecticide resistance and may inform the development of novel mosquito control strategies.

## Methods

### Mosquito collection and rearing

The *Aedes albopictus* laboratory-susceptible strain was donated by Southern Medical University and maintained at Hainan Medical University for 6 years. The field *Ae. albopictus* larvae were collected from June 2022 to October 2023 (Haikou City, Ledong City, Ding’an County, Wenchang City, Danzhou City) (Table S1). Larvae were reared in the insectary at Hainan Medical University under controlled conditions with a temperature of 26 ± 2 °C, a relative humidity of 60 ± 10%, and a photoperiod of 12:12 h (light:dark). Larvae were fed with a mixture (1:4) of yeast powder and fish food. Emerged adult mosquitoes were transferred to the mosquito cages and fed with a 10% glucose solution. Nonblood-fed F0 to F1 generation female mosquitoes were used for insecticide resistance bioassays.

### WHO insecticide susceptibility bioassays

Adult insecticide susceptibility assays were conducted following WHO standard protocols [27, 28]. For adult assays, 3–4 day-old unfed females were exposed to standard 0.03% deltamethrin impregnated test paper in WHO tube bioassays for 60 min knockdown exposure, followed by recovery period in untreated tubes. Mortality was recorded at 24 h post-exposure. Knockdown rates were recorded every 10 min within the first 60 min, and 24 h mortality rates were recorded for each replicate. Resistance classification followed WHO criteria: resistant (< 90% mortality), probably resistant (90–98% mortality), and susceptible (> 98% mortality) [28]. Deltamethrin test and control papers were supplied by the School of Biological Sciences, Universiti Sains Malaysia (Penang, Malaysia).

### DNA extraction and bacteria identification

Genomic DNA from single bacterial colonies was extracted following the instructions of the Sangon Biotech^®^ rapid bacterial genomic DNA isolation kit protocols. The *16S* rRNA gene was amplified using primer 27F (5′-AGAGTTTGATCCTGGCTCAG-3′) and 1492R (5′-TACGGCTACCTTGTTACGACTT-3′) [29]. The PCR reaction conditions were 95 °C for 5 min; 95 °C for 30 s, 55 °C for 30 s, 72 °C for 1 min 30 s, with 30 cycles; and a final extension at 72 °C for 10 min in a reaction volume of 25 µL. The PCR products were sent to the Beijing Genomics Institute for double strand reading, and the obtained sequences were spliced by SeqMan software.

### Screening of gut bacteria related to deltamethrin resistance in *Aedes albopictus*

**Fig. 1:**
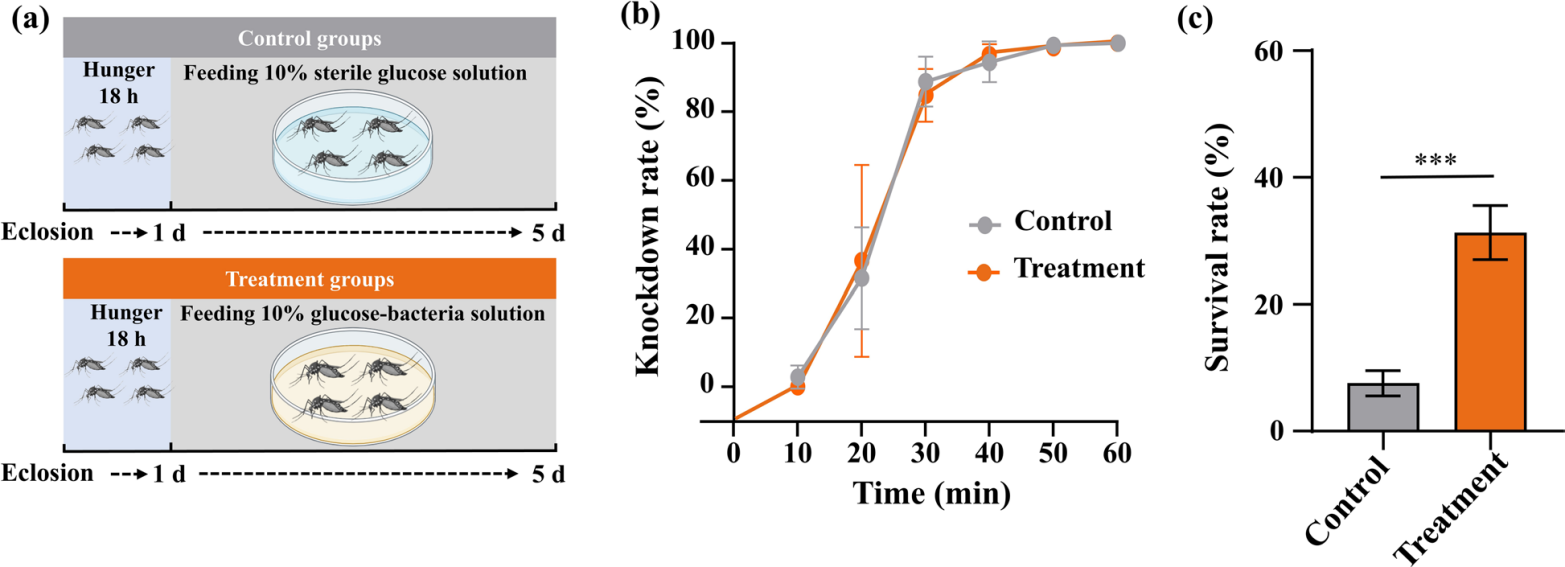
Enhanced deltamethrin resistance following *B. cereus* infection in laboratory-susceptible *Ae. albopictus*. (**a**) Experimental design showing mosquito infection protocol. One day post eclosion *Ae. albopictus* mosquitoes were starved for 18 h, then control and treatment groups were fed 10% sterile glucose solution and 10% glucose–bacteria solution, respectively, for 5 days. (**b**) Knockdown rates and (**c**) survival rates of *Ae. albopictus* after exposure to 0.015% deltamethrin after the 24 h recovery period. ^***^P < 0.005

Twenty-five gut bacteria species previously isolated and purified from field-collected deltamethrin-resistant larvae of *Ae. albopictus* [26] were cultured overnight in Luria–Bertani (LB) medium at 170 rpm and 37 °C. In total, 5 ml of bacterial culture, adjusted to an OD_600_ of 0.5, were used to prepare the glucose–bacterial solution (GBS). The cultures were centrifuged at 3500 × *g* for 10 min, and the bacterial pellets were washed twice with sterile water through repeated centrifugation and resuspension. The final pellets were then mixed with 5 mL of 10% sterile glucose solution to obtain the GBS. The treatment groups of laboratory-susceptible adult mosquitoes (1 day after eclosion in the starving state) were continuously fed with sterile cotton dipped of GBS for 5 days, while the control groups were fed with a 10% sterile glucose solution for 5 days (Fig. 1a). The cotton pieces were replaced three times a day to minimize the potential of bacterial contamination from the air.

Adult mosquitoes infected with bacteria (25 bacterial species isolated from deltamethrin-resistant larvae of *Ae. albopictus*) were assigned to the treatment group, and were subjected to WHO deltamethrin susceptibility bioassays as described above with appropriate modifications (using 0.015% deltamethrin impregnated test paper) following the method of Wang et al. [30]. Six replicates of 25 female mosquitoes underwent the bioassays. In parallel, control groups of untreated laboratory-susceptible mosquitoes underwent identical bioassays. Differential survival rates between bacterial-infected and control groups indicated resistance-enhancing bacterial candidates.

On the basis of the preliminary analysis of resistance test results, feasibility for easy culturing, and feeding success, *B. cereus*_HL4.2 was selected for further study and analysis to examine its impact on the resistance of *Ae. albopictus* to deltamethrin. The *B. cereus*_HL4.2 *16S* rRNA sequencing data have been deposited in the NCBI with the accession number PP572860. *Bacillus cereus* is a Gram-positive, beta-hemolytic, rod-shaped, pore-forming, facultatively anaerobic bacterium. It is mobile and lacks capsules. Colonies are large, with rough, flattened, irregular surfaces. In the LB plate medium, 37 °C, culture for 24 h, it forms round, soft-textured, non-pigmented, slightly glossy white colonies (a candle-like color) (Fig. S1a, b).

### Quantification of *Bacillus cereus*_HL4.2 colonization in *Aedes albopictus*

Both PCR and quantitative PCR (qPCR) were employed for qualitative and quantitative analysis of *B. cereus*_HL4.2 abundance, respectively. Genomic DNA was extracted from individual female mosquitoes (*N* = 20 per group) using the phenol–chloroform method after lysozyme lysis [31]. The presence of *B. cereus*_HL4.2 was confirmed using the *gyrB* gene as a marker [32] (Table S2). PCR amplification was carried out in a 25 µL reaction volume under the following conditions: initial denaturation at 95 °C for 15 min; 35 cycles of 95 °C for 30 s, 60 °C for 1 min, and 72 °C for 30 s; and a final extension at 72 °C for 2 min.

For quantitative analysis, the target *gyrB* gene was cloned into the pLB vector and transformed into *Escherichia coli* DH5α competent cells using the TIANGEN Fast Cloning Kit (cat. no. VT205). Recombinant cells were cultured overnight in 5 mL LB broth supplemented with 100 μg/mL ampicillin at 37 °C and 170 rpm. The plasmids (pLB-*gyrB*) were extracted and validated by sequencing at BGI. To generate a standard curve, pLB-*gyrB* plasmids with known copy numbers were serially diluted tenfold (10^−1^ to 10^−9^). DNA from mosquito samples and the dilution series were used as templates for qPCR with SuperReal PreMix Plus (cat. no. FP217). Ct values were recorded, and the corresponding number of copies per mosquito were determined.

### Impact of *Bacillus cereus*_HL4.2 on mosquito fitness and fecundity in *Aedes albopictus*

To evaluate the potential fitness costs of *B. cereus*_HL4.2 colonization, life-table experiments were conducted using laboratory-susceptible *Ae. albopictus* mosquitoes that were orally infected with the glucose–bacterial solution (GBS) three times daily for five consecutive days, while control mosquitoes were fed with sterile glucose solution alone. The experiments were conducted with eight replicates and ten females for each replicate.

To examine the impact of *B. cereus*_HL4.2 infection on the fecundity of *Ae. albopictus*, we used mice to feed the treatment and control laboratory-susceptible female *Ae. albopictus*. Three-day-old females were allowed to feed on mice blood for 3 h and then maintained on a 10% sugar solution. After 2 days of blood-feeding, females were put in cages with oviposition water cups inside. In the 500-mL water cup, a piece of filter paper towel lined with the cup with direct contact to the water and the air interface to allow for female to lay eggs. After 3 days, the paper towel was removed. The eggs on the filter paper were counted under the microscope. The experiments were conducted with eight replicates with ten females for each replicate. The experiments were conducted for both treatment and control groups.

### Detection of *Bacillus cereus* abundance in the field *Aedes albopictus*

To examine whether natural *B. cereus* prevalence correlates with insecticide resistance phenotypes in field populations, we conducted resistance screening and qPCR quantification of *B. cereus* abundance in *Ae. albopictus* populations collected from five different study sites where resistance levels varied. The field-collected *Ae. albopictus* larvae from different sites (Haikou, Wenchang, Ledong, Dingan, and Danzhou) were reared in the laboratory until adult mosquito emergence. Emerged adult mosquitoes were fed with a 10% glucose solution. Three- to five-day-old female mosquitoes were tested for their resistance to 0.03% deltamethrin using the WHO tube test. Resistance assays were performed with four replicates per site, each consisting of 25 females. Simultaneously, *B. cereus* abundance in the gut of 3- to 5-day-old female *Ae. albopictus* was quantified by qPCR using ten females from each study site.

### *Bacillus cereus*_HL4.2 reinfection in field-collected *Aedes albopictus* after antibiotic gut clearance

To examine if infection of *Ae. albopictus* with *B. cereus*_HL4.2 affects mosquito resistance to deltamethrin, field-collected resistant *Ae. albopictus* (Haikou, Dingan, and Ledong) were treated with gentamicin (150 μg/L) and streptomycin (150 μg/L) mixed with 10% sterile glucose for 2 days and then switched to 10% sterile glucose solution for 3 days (control) or gentamicin (150 μg/L) and streptomycin (150 μg/L) mixed with 10% sterile glucose for 2 days, and then switched to a mixed solution of *B. cereus*_HL4.2–10% sterile glucose for 3 days (treatment) (Fig. 5a). The two groups were treated simultaneously and tested for resistance against 0.03% deltamethrin as previously described, with five biological replicates of 25 females each.

### Mosquito detoxifying enzyme activities before and after *Bacillus cereus*_HL4.2 infection

Activities of three detoxification enzymes were measured in both laboratory and field populations of *Ae. albopictus* (Haikou, Dingan, and Ledong) before and after infection with *B. cereus*_HL4.2: cytochrome P450 monooxygenases (P450s), glutathione *S*-transferases (GSTs), and carboxylesterases (COEs), following the treatment procedure described above. The measurement of P450 and GST activity was conducted in accordance with the methodologies proposed by Penilla et al. [33] and Zhong et al. [34], respectively. The COE activity was quantified according to the methodology of Hosokawa and Satoh [35]. Total protein was quantified for each mosquito using the method of Bradford [36]. Mean absorbance values for each tested mosquito and enzyme were converted into enzyme activity and normalized on the basis of the total protein amount. P450 and GST activities were calculated as pmol 7-HC/min/mg protein and μmol cDNB/min/mg protein, respectively. The COE activity was calculated as μmol p-nitrophenol/min/mg protein, using the formula (Δabsorbance/min−Δblank/min) × 1.0/16.4 × 0.05 × protein (mg/mL). An absorption coefficient of 16,400 /M/cm was employed [37]. All measurements were conducted in duplicate. Thirty 3–5-day-old adult females were tested for each group.

### RNA-seq and differential genes validation before and after *Bacillus cereus*_HL4.2 infection

To investigate whether *B. cereus*_HL4.2 reduces deltamethrin susceptibility in *Ae. albopictus* by inducing the expression of metabolic detoxification-related genes, we compared the transcriptomic profiles of mosquitoes before and after *B. cereus*_HL4.2 enrichment. The laboratory-susceptible *Ae. albopictus* mosquitoes before and after infection with *B. cereus*_HL4.2 were sent to the Beijing Genomics Institute (BGI) for transcriptome sequencing. Total RNA was extracted from two groups of mosquito samples before and after infection with *B. cereus*_HL4.2 using the Tiangen RNA Simple Total RNA Extraction Kit (cat. no. DP419), with five biological replicates of 20 female mosquitoes each. The RNA-seq raw data obtained from sequencing were filtered using SOAPnuke (v1.5.6) [38], which filtered out reads containing junctions (junction contamination), reads with unknown base N content greater than 5%, and low-quality reads (reads with a quality value of fewer than 15 bases accounting for more than 20% of the total number of bases in the reads were considered low-quality reads) to obtain clean data. Reads were aligned to the *Ae. albopictus* reference genome using Bowtie2 [39]. Transcript quantification was performed using RSEM (v1.3.1) [40], and differential gene expression analysis employed DESeq2 (v1.4.5) [41] with a *Q* value of ≤ 0.05 or FDR ≤ 0.001. To explore the gene functions associated with phenotypic changes in further depth, we analyzed the GO (http://www.geneontology.org/) and KEGG (https://www.kegg.jp/) enrichment of differential genes using Phyper based on the hypergeometric test with *Q* values ≤ 0.05 as a threshold, and satisfying this condition was defined as significant enrichment in candidate genes.

### Real-time (RT)-PCR validation of differentially expressed genes

The differentially expressed genes (DEGs) of *Ae. albopictus* in the transcriptome sequencing results were validated with |log_2_fold change|> 1 and designing primer sequences for the coding sequence of the differential gene using Oligo7 (v.7.37) (Table S2). The extracted total RNA of laboratory *Ae. albopictus* before and after infection with *B. cereus*_HL4.2 was reverse transcribed using Tiangen FastKing RT Kit (cat. no. KR116). Subsequently, the reverse-transcribed samples were subjected to real-time PCR by Takara TB Green^®^ Premix Ex Taq^™^ II (cat. no. RR820A). Finally, relative quantitative ΔCt and 2^−ΔΔCt^ were used to calculate the differential multiples.

### In vitro assay of deltamethrin degradation by *Bacillus cereus*_HL4.2

To verify the degradation ability of *B. cereus*_HL4.2 on deltamethrin, we followed established protocols in previous studies [30]. Firstly, 0 mg/L, 50 mg/L, 100 mg/L, and 200 mg/L solutions of deltamethrin were prepared by dissolving deltamethrin in acetone. Subsequently, 100 μL of each of the different concentrations of deltamethrin solution was uniformly spread on Hope Bio inorganic salt solid plates (cat. no. HB8761). Then, 60 μL of *B. cereus_*HL4.2 suspension was evenly spread onto the plates. This suspension was prepared from overnight LB broth cultures adjusted to an OD_600_ of 0.5. The cultures were centrifuged, and the resulting bacterial pellet was resuspended in sterile normal saline (0.9% sodium chloride) to the desired concentration. All plates were incubated in an aerobic incubator at 37 °C for 24 h and each concentration was repeated three times to observe the growth of colonies.

### GFP tagging of *Bacillus cereus*_HL4.2 for persistence tracking

To track *B. cereus*_HL4.2 colonization and persistence through mosquito development, we constructed a green fluorescent protein (GFP)-expressing strain. For this purpose, a plasmid (pUC19-GFP; purchased from Shanghai Baosai Biotechnology) that simultaneously expresses green fluorescent protein and chloromycetin resistance (10 μg/mL) was used. After recovery of *B. cereus*_HL4.2, it was rinsed 2–3 times with sterile deionized water and centrifuged at 5000 × *g* at 4 °C. The organisms were collected and prepared as *B. cereus*_HL4.2 competent cells.

The 5 μL of GFP plasmid was electroshock transformed into 50 μL of *B. cereus*_HL4.2 competent cells under the following transformation conditions: the 0.2 cm shock cup was inserted into the shock groove, and the voltage of 1600 V, resistance of 200 Ω, and capacitance of 25 μF were set to perform the shock, and the shock conversion time was 4.6 ms.

The transformed GFP-*B. cereus*_HL4.2 was resuspended using normal saline (0.9% sodium chloride) and subsequently mixed with fish feed and fed to laboratory *Ae. albopictus* larvae. Mosquito larvae were exposed to B. cereus infection by feeding on fish food supplemented with bacterial cells obtained from 5 mL of LB broth culture grown to an OD_600_ of 0.5 and subsequently centrifuged. The bacteria-supplemented food was provided for 24 h, after which the green fluorescence expression was observed in larvae, pupae, and adult mosquitoes under a fluorescence microscope.

### Statistical analysis

Resistance classification of adult mosquitoes following WHO criteria: resistant for < 90% mortality, probable resistance for 90–98% mortality, and susceptible for > 98% mortality [28]. For normally distributed data a Student’s *t*-test was used; for non-normally distributed data the Mann–Whitney *U* test was applied. These tests were used to compare knockdown rate, survival rate, *B. cereus*_HL4.2 abundance in the mosquito gut, egg laying amount, emergence rate, adult mosquito survival time, and average number of eggs laid per female during its lifetime, as well as the activities of P450s, GSTs, and COEs between control and treatment groups. In the larval bioassay, the median lethal concentration (LC_50_) and the corresponding 95% confidence intervals were calculated on the basis of the recorded data using Schoofs and Willhite’s probit analysis with SPSS Statistics (v27.0, IBM, Chicago, IL, USA) [42]. One-way analysis of variance (ANOVA) with post hoc Tukey honestly significant difference (HSD) test was used to determine the differences in *B. cereus* copy number in mosquitoes from the different study areas. Adult survivorship was evaluated using Kaplan–Meier survival analysis [43]. The log-rank test was used to compare the difference in survival curves between *B. cereus* control and treatment groups. All analyses were performed using JMP Pro 17 statistical software (JMP, SAS Institute Inc., Cary, NC, USA) or SPSS (vR27.0) (IMB, Chicago, IL, USA) statistical software, and *P* < 0.05 was considered statistically significant.

## Results

### Reduced deltamethrin susceptibility in *Aedes albopictus* after oral infection of* Bacillus cereus*_HL4.2

Among 25 bacterial isolates obtained from deltamethrin-resistant *Aedes albopictus*, *B. cereus*_HL4.2 was identified as being potentially associated with reduced deltamethrin susceptibility. Oral feeding of laboratory-susceptible *Ae. albopictus* with *B. cereus*_HL4.2-containing sucrose medium significantly elevated survival rates following exposure to deltamethrin-impregnated filter paper (Fig. 1a). After a 1 h exposure to 0.015% deltamethrin test paper, both *B. cereus* infected and noninfected *Ae. albopictus* mosquitoes were completely knocked down (Fig. 1b). However, after 24 h of recovery, the survival rate of *Ae. albopictus* infected with *B. cereus*_HL4.2 increased significantly from 7.6 (± 2.0 SE) % to 31.3 (± 4.3 SE) % (Student’s *t*-test: *t*_(11)_ = 5.046, *P* < 0.001) (Fig. 1c). These results indicate that oral infection with *B. cereus*_HL4.2 significantly reduces the susceptibility of *Ae. albopictus* to deltamethrin, leading to an enhanced survival rate following insecticide exposure.

### Confirmation of successful oral infection with *Bacillus cereus*_HL4.2

**Fig. 2:**
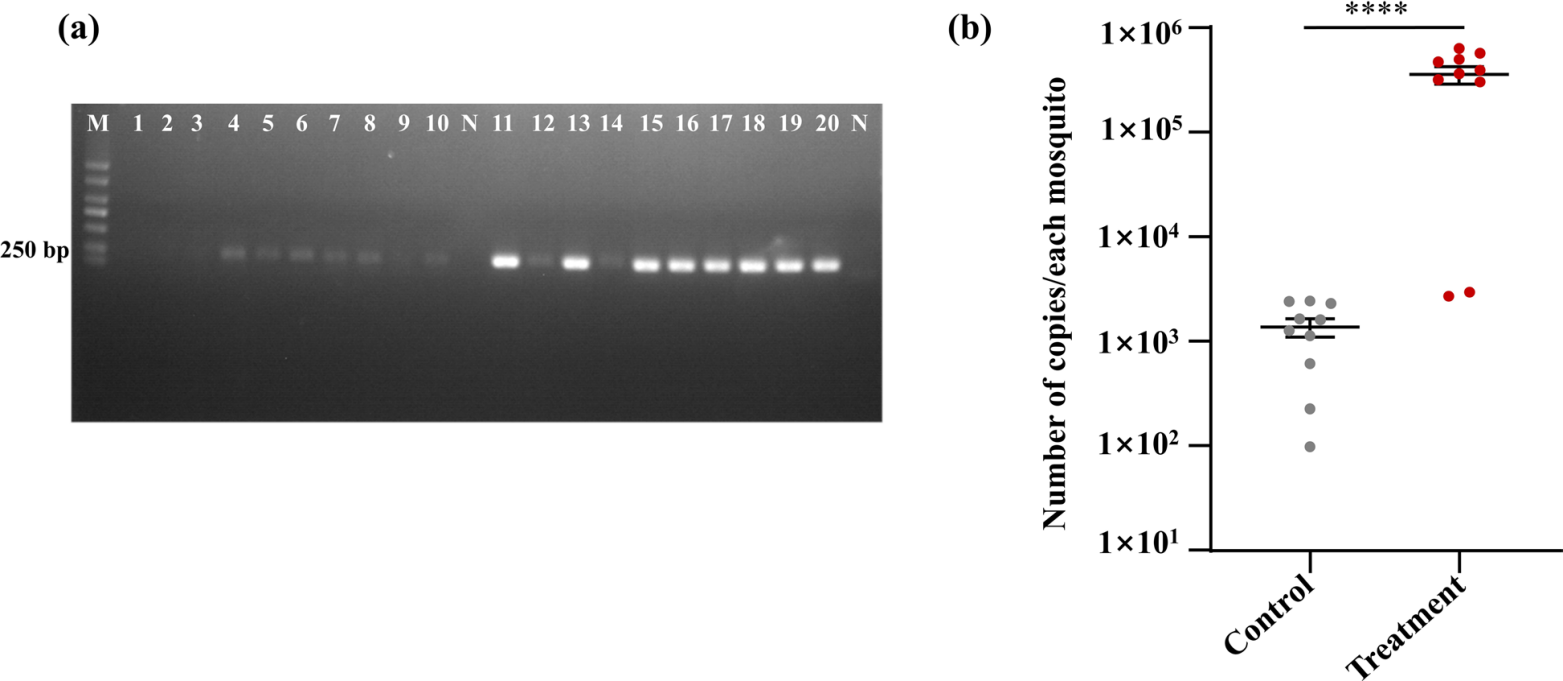
Verification of successful *B. cereus* infection in laboratory-susceptible *Ae. albopictus* gut after 5 days of oral feeding. (**a**) PCR detection of *B. cereus*. Templates included control mosquito gut samples (lanes 1–10), *B. cereus-*infected gut samples (lanes 11–20), DL2000 maker (lane M), and negative control (lanes N). (**b**) Quantification of *B. cereus* abundance in mosquito guts using qPCR, expressed as copy number per mosquito. ^****^P < 0.0001

Having established that *B. cereus* infection reduces deltamethrin susceptibility, we next verified successful bacterial colonization of *B. cereus* abundance in the gut of infected laboratory-susceptible strains of *Ae. albopictus* after 5 days of oral feeding. PCR analysis revealed that the amplicon from *gyrB* gene detected in the infected group was much greater than that of the control group, suggesting a higher bacterial load in the treatment groups (Fig. 2a). Quantitative qPCR further demonstrated that the abundance of *B. cereus* in the mosquito gut was significantly higher in the treatment groups (362,978.97 ± 69,015.4 copies per mosquito) compared with the control groups (1397.51 ± 279.76 copies per mosquito) (Student’s *t*-test: *t*_(18)_ = 5.239, *P* < 0.05) (Fig. 2b). This approximately 260-fold increase in *B. cereus* abundance confirms successful establishment of the bacterium in the mosquito gut following oral feeding.

### *Bacillus cereus*_HL4.2 infection does not impose fitness costs on *Aedes albopictus*

**Fig. 3:**
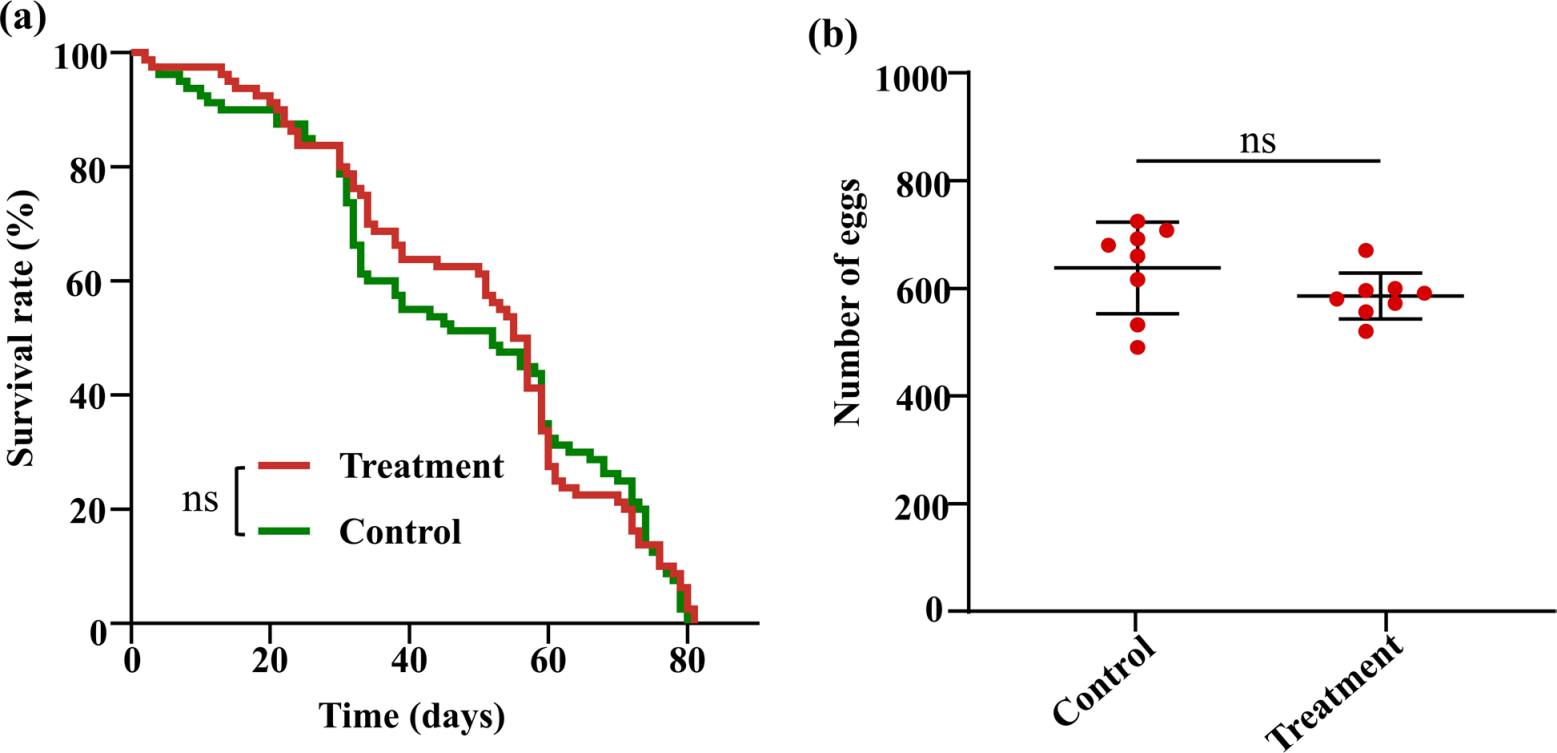
Impact of *B. cereus* infection on laboratory-susceptible *Ae. albopictus* fitness parameters. (**a**) Adult mosquito survival curves for control and *B. cereus*-infected groups. (**b**) Egg production per ten females following blood feeding in control and *B. cereus*-infected groups. ns represents no significant differences (*P* > 0.05)

To determine whether *B. cereus*_HL4.2 colonization incurred any fitness penalties that might limit its epidemiological relevance, we conducted life-table experiments on *B. cereus*_HL4.2-infected versus control laboratory-susceptible females. Survival studies revealed no significant difference in adult lifespan between *B. cereus*_HL4.2-infected *Ae. albopictus* (50.1 ± 2.3 days) and control groups (48.0 ± 2.5 days) (log-rank tests, *χ*^*2*^ = 0.167, d.f. = 1, *P* > 0.05) (Fig. 3a). Similarly, fecundity was unaffected, with no significant difference in egg production between treatment groups (585.6 ± 15.2 eggs per ten females) and control groups (637.8 ± 30.2 eggs per ten females) following blood feeding (Student’s *t*-test: *t*_(14)_ = −1.543, *P* = 0.153) (Fig. 3b).

### Positive association between field *Bacillus cereus*_HL4.2 abundance and deltamethrin resistance

**Fig. 4:**
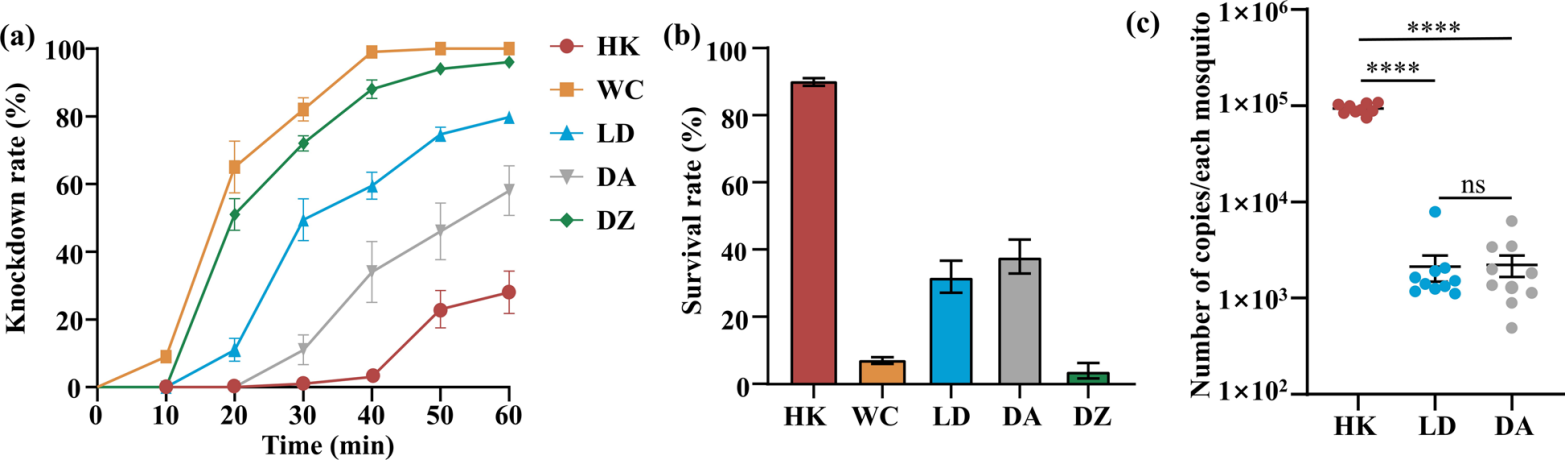
Correlation between deltamethrin resistance and *B. cereus* abundance in field populations of *Ae. albopictus* from Hainan Province. (**a**) Knockdown rate and (**b**) survival rate of field-collected mosquitoes from different locations. (**c**) Abundance of *B. cereus* in the gut of field-collected *Ae. albopictus* from Haikou (HK), Wenchang (WC), Ledong (LD), Ding’an (DA), and Danzhou (DZ). ^****^P < 0.0001. ns indicates no significant differences (*P* > 0.05)

To evaluate the ecological relevance of the laboratory findings, we examined whether natural *B. cereus* prevalence correlates with population-level deltamethrin resistance phenotypes across five field sites in Hainan Province. The survival rates of the field mosquitoes exposed to deltamethrin varied considerably: 89.3 (± 1.2 SE) % in Haikou, 38.0 (± 4.4 SE) % in Ding’an, 32.0 (± 2.3 SE) % in Ledong, 7.0 (± 1.2 SE) % in Wenchang, and 4.0 (± 2.3 SE) % in Danzhou (Fig. 4a, b). Pairwise comparisons illustrate that these five populations could be classified into three distinct resistance groups: high resistance (Haikou), moderate resistance (Ding’an and Ledong), and low resistance (Wenchang and Danzhou). Strikingly, quantitative detection of *B. cereus* in the gut of *Ae. albopictus* showed that mosquitoes from Haikou harbored significantly higher quantities of *B. cereus* (93,612.1 ± 3817.2 copies/mosquito) compared with populations from Ledong (2130.7 ± 646.6 copies/mosquito) and Ding’an (2221.0 ± 555.3 copies/mosquito), with no significance in *B. cereus* quantity between the latter two sites (ANOVA, *F*(_2, 26_) = 605.3, *P* < 0.0001; Tukey HSD, *P* < 0.0001) (Fig. 4c). This correlation between natural *B. cereus* abundance and deltamethrin resistance in field populations further supports the hypothesis that this bacterium potentially modulates insecticide susceptibility.

### *Bacillus cereus*_HL4.2 infection restores deltamethrin resistance in antibiotic-treated* Aedes albopictus*

**Fig. 5:**
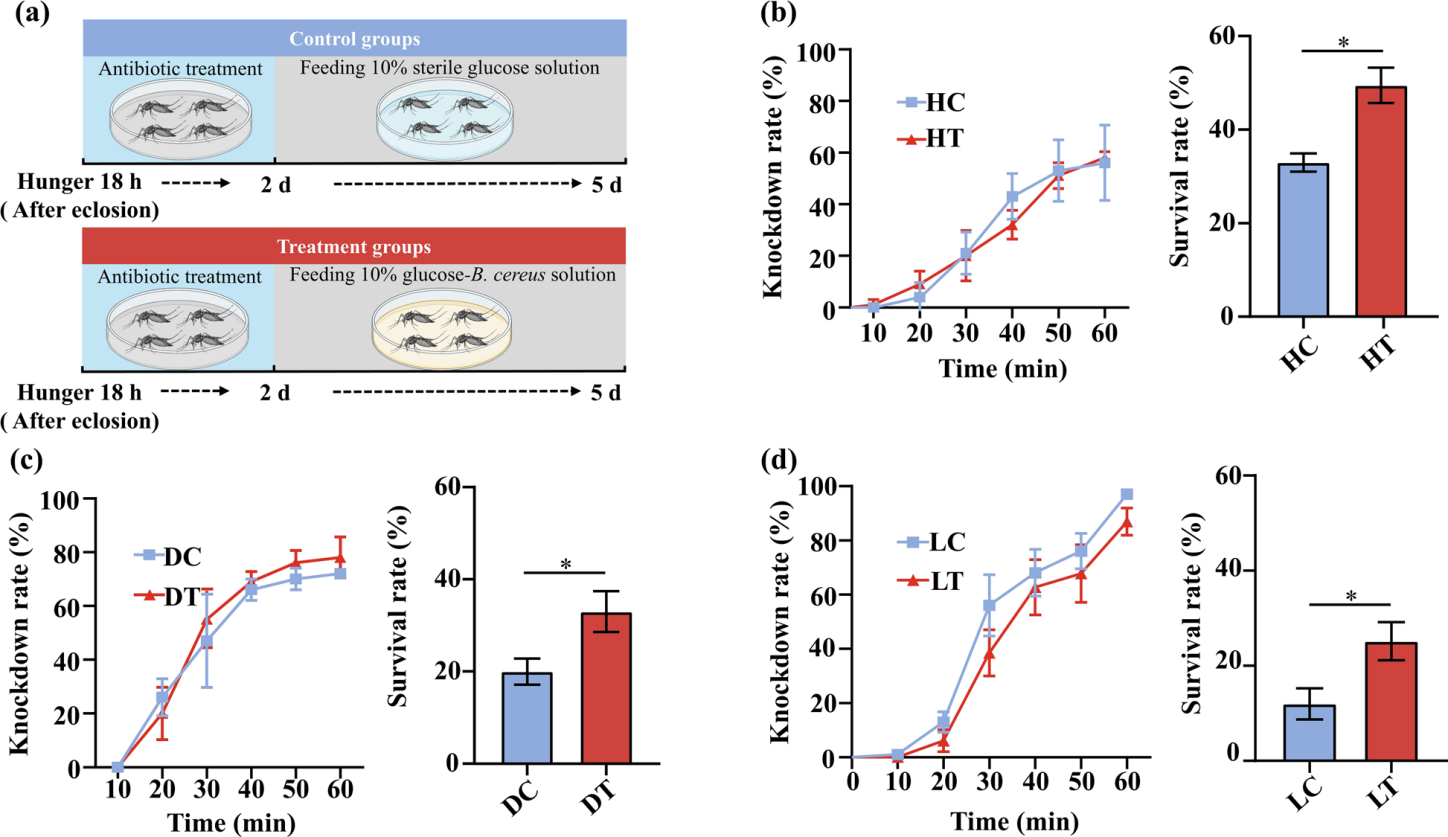
Restoration of deltamethrin resistance through *B. cereus* reinfection after antibiotic treatment in field *Ae. albopictus* mosquitoes. (**a**) Experimental design: field-collected *Ae. albopictus* after eclosion (hunger 18 h) were treated with antibiotics for 2 days followed by either 10% sterile glucose solution (control groups) or 10% glucose–*B. cereus* solution (treatment groups) for 3 days. Changes in knockdown rates and survival rates after deltamethrin exposure in: (**b**) Haikou, (**c**) Ding’an, and (**d**) Ledong populations. HC/HT, Haikou control/treatment groups; DC/DT, Ding’an control/treatment groups; LC/LT, Ledong control/treatment groups. ^*^P < 0.05

To further demonstrate the causal relationship between *B. cereus*_HL4.2 and deltamethrin resistance, we conducted an antibiotic clearance and reinfection experiment using field-collected mosquitoes. Following antibiotic treatment to reduce native gut microbiota and subsequent reinfection with *B. cereus*_HL4.2 (Fig. 5a; Fig. S2), we observed significantly higher survival rates in the treatment groups compared with control groups across all three field populations tested: Haikou (49.5 ± 3.8% SE versus 33.0 ± 1.9% SE; Student’s *t*-test, *t* = 3.90, *P* < 0.05) (Fig. 5b), Ding’an (33.0 ± 4.4% SE versus 20.0 ± 2.8% SE; Student’s *t*-test, *t* = 2.47, *P* < 0.05) (Fig. 5c), and Ledong (25.3 ± 3.9% SE versus 12.0 ± 3.3% SE; Student’s *t*-test, *t* = 2.59, *P* < 0.05) (Fig. 5d). These results provide evidence that *B. cereus*_HL4.2 directly contributes to deltamethrin resistance in *Ae. albopictus*.

### *Bacillus cereus*_HL4.2 promotes detoxification enzyme activities in *Aedes albopictus*

**Fig. 6:**
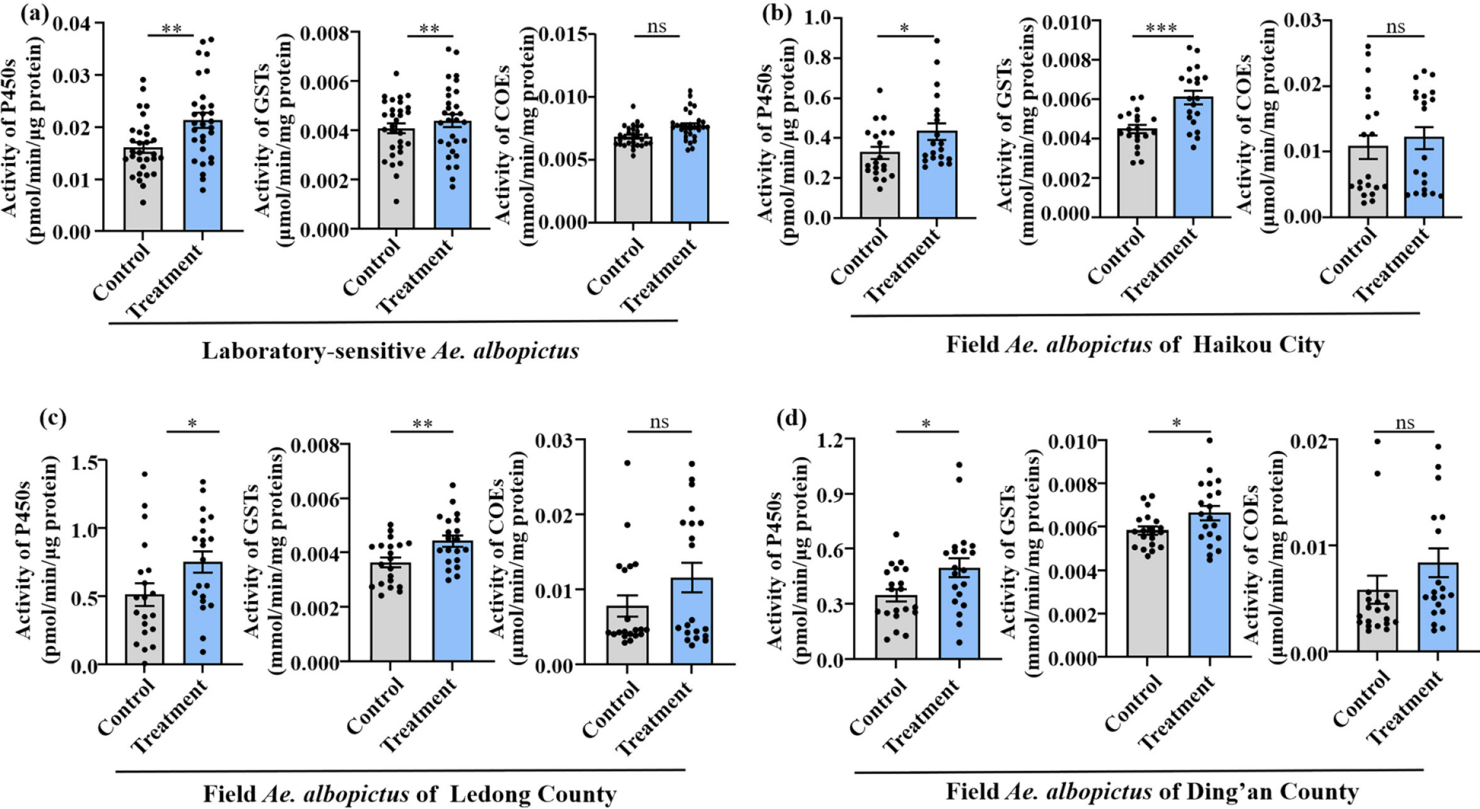
Alterations in three detoxification enzyme activities of *Ae. albopictus* before and after infection with *B. cereus.* (**a**) Laboratory-susceptible strain. (**b**) Haikou field population. (**c**) Ledong field population. (**d**) Ding’an field population. ^*^P < 0.05, ^**^P < 0.01, and ^***^P < 0.005. ns represents no significant differences (*P* > 0.05)

To investigate the potential mechanisms underlying *B. cereus*_HL4.2-mediated deltamethrin resistance, we examined the activities of three major detoxification enzyme families in infected and control mosquitoes. Compared with regular feeding with sugar solutions, oral feeding with *B. cereus*_HL4.2 significantly increased both P450 and GST enzyme activities in both laboratory strains (1.39- and 1.21-fold) and field-collected populations of *Ae. albopictus* (Student’s *t*-test, all *P* < 0.05; Fig. 6a–d). However, adding *B. cereus*_HL4.2 did not significantly affect carboxylesterase (COE) enzyme activities in any of the mosquito populations tested (Student’s *t*-test, all* P* > 0.05; Fig. 6a–d). This selective enhancement of P450 and GST activities, both crucial for pyrethroid detoxification, provide a biochemical basis for enhanced detoxification of deltamethrin.

### Transcriptomic profiling reveals upregulation of metabolic detoxification pathways in *Aedes albopictus*

**Fig. 7:**
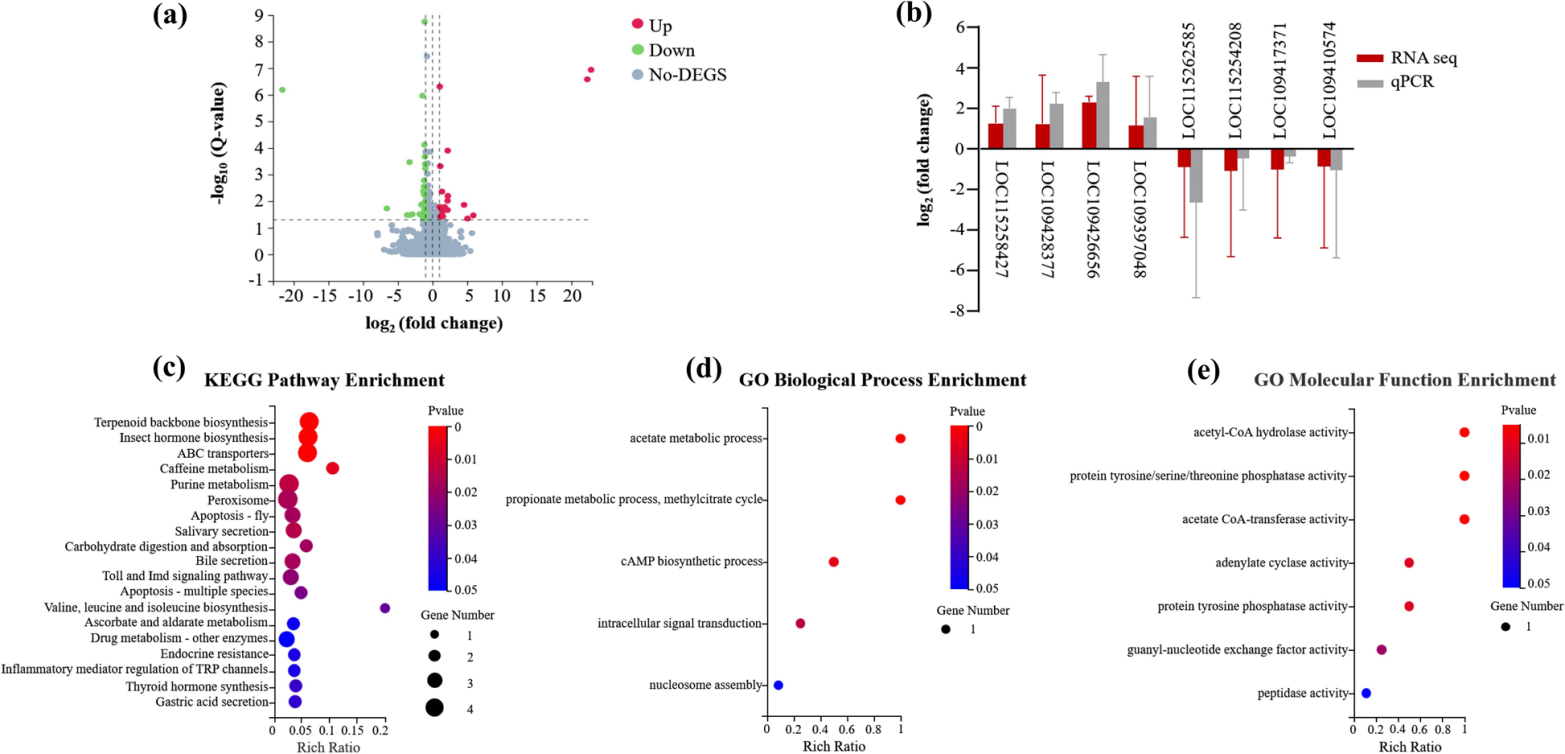
Transcriptomic changes in laboratory-susceptible *Ae. albopictus* following *B. cereus* infection. (**a**) Volcano plot of differentially expressed genes (DEGs). Red points represent upregulated DEGs, green points represent downregulated DEGs, and gray points represent non-DEGs. (**b**) Expression validation of selected DEGs (|log_2_ fold change|> 1) using qPCR. (**c**) KEGG pathway enrichment analysis showing significantly altered pathways. (**d**) GO biological process and (**e**) GO molecular function enrichment analysis

To further explore the molecular mechanism underlying *B. cereus*_HL4.2-mediated resistance, we performed RNA sequencing of *Ae. albopictus* before and after infection of *B. cereus*_HL4.2. A total of 122 differentially expressed genes (DEGs) were identified, with 31 upregulated and 91 downregulated genes in infected mosquitoes (Fig. 7a). RT-qPCR validation confirmed the differential expression patterns of selected genes with |log_2_ fold change|> 1. Four significantly upregulated and four significantly downregulated genes were selected for RT-qPCR validation. The upregulated genes included *uncharacterized LOC109417371*, *uncharacterized LOC115262585*, *angiopoietin-4-like*, and *ficolin-3-like*, with relative expression levels of 3.82, 3.82, 3.39, and 2.87, respectively. The downregulated genes included *uncharacterized LOC109397048*, *perlucin-like*, *pancreatic lipase-related protein 2-like*, and, *-threonine ammonia-lyase-like*, with relative expression levels of 0.43, 0.13, 0.35, and 0.67, respectively. The RT-qPCR results were consistent with the RNA-seq findings (Fig. 7b; Table S3). A subset of genes associated with the cAMP signaling pathway and purine metabolism was significantly upregulated. Conversely, genes enriched in ABC transporters and sensory system pathways were predominantly downregulated (Fig. 7c; Table S4). GO enrichment analysis identified significant enrichment of DEGs involved in acetyl-CoA hydrolase activity, acetate CoA-transferase activity, protein tyrosine/serine/threonine phosphatase activity, acetate metabolic processes, and cAMP biosynthetic processes (Fig. 7d, e; Table S5). These transcriptomic changes suggest that *B. cereus*_HL4.2 infection alters multiple metabolic and signaling pathways in *Ae. albopictus*, potentially contributing to the reduced susceptibility to deltamethrin.

### *Bacillus cereus*_HL4.2 could directly degrade deltamethrin in vitro

In addition to modifying host physiology, we investigated whether *B. cereus*_HL4.2 could directly degrade deltamethrin. When cultured on inorganic salt medium with different concentrations of deltamethrin as the sole carbon source, *B. cereus*_HL4.2 colony abundance showed a positive correlation with deltamethrin concentrations (Fig. S3). The number of colonies formed on 0 mg/L, 50 mg/L, 100 mg/L, and 200 mg/L deltamethrin–inorganic salt medium was 0 CFU, 295.7 ± 14.6 CFU, 669.0 ± 30.7 CFU, and 1160.7 ± 34.8 CFU, respectively (Fig. S3). This dose-dependent growth pattern provides strong evidence that *B. cereus*_HL4.2 can utilize deltamethrin as a nutrient source, suggesting direct degradation of the insecticide as an additional resistance mechanism.

### *Bacillus cereus*_HL4.2 persists throughout *Aedes albopictus* development

**Fig. 8:**
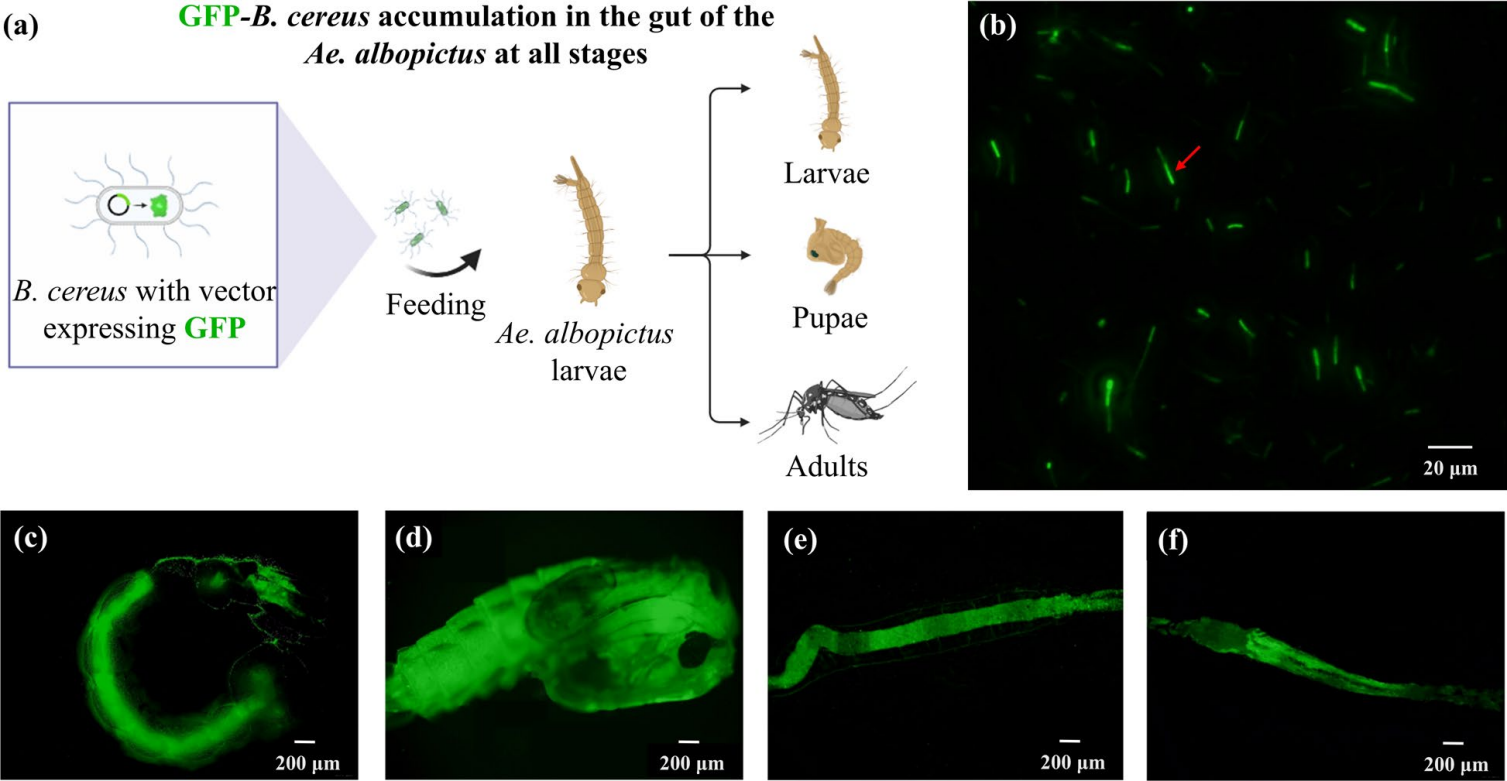
Accumulation of GFP-tagged *B. cereus* throughout *Ae. albopictus* developmental stages. (**a**) Experimental scheme for tracking GFP-*B. cereus* in *Ae. albopictus* development. (**b**) GFP-*B. cereus* under the fluorescence microscope. (**c**) GFP-*B. cereus* in larval whole-body. (**d**) GFP-*B. cereus* in pupal whole-body. (**e**) GFP-*B. cereus* in larval gut. (**f**) GFP-*B. cereus* in adult mosquito gut. Red arrows represent GFP-tagged *B. cereus*

To determine the epidemiological relevance of *B. cereus*_HL4.2 to field populations, we tracked bacterial persistence through the mosquito developmental cycle. Following transformation with a pUC19-GFP fluorescent plasmid, GFP-expressing *B. cereus*_HL4.2 was fed to *Ae. albopictus* larvae (Fig. 8a, b). Green fluorescence was subsequently observed in the gut of larval, pupal, and adult stages, confirming that *B. cereus*_HL4.2 persists throughout mosquito metamorphosis (Fig. 8c–f). However, no fluorescence was detected in the eggs in the next generation (data not shown), indicating that vertical transmission does not occur. These findings suggest that while *B. cereus*_HL4.2 is not vertically transmitted, it can provide continuous protection against insecticides throughout the mosquito’s lifespan once acquired in the larval stage.

## Discussion

Insecticide resistance poses an escalating threat to mosquito vector control and arthropod-borne disease prevention globally [44–46]. Recent reports have shown that *Ae. albopictus* populations in China have rapidly increased their resistance to pyrethroid insecticides [47–49]. Several studies have suggested that gut microbes may play an important regulatory role in insect insecticide resistance [23, 50]. For example, Chen et al. demonstrated that the gut symbiont *Citrobacter* sp. (CF-BD) plays a key role in the degradation of trichlorfon in the oriental fruit fly *Bactrocera dorsalis* (Hendel) [51]. Similarly, Scathes et al. found that adding cultured gut bacteria isolated from mosquito larvae to antibiotic-cleared larvae food significantly reduced *Ae. aegypti* larval mortality against propoxur larvicides [52]. However, few studies have directly investigated the mechanisms by which bacteria influence mosquito resistance to insecticides. In this study, we demonstrated an association between the gut symbiotic bacterium *B. cereus*_HL4.2 and reduced deltamethrin susceptibility in *Ae. albopictus*, supported by multiple complementary experimental approaches. Using a series of carefully designed experiments, we established that oral infection with *B. cereus*_HL4.2 increases mosquito survival following deltamethrin exposure, and that this protective effect occurs without any detectable fitness costs to the host on the basis of the parameters investigated. Importantly, we observed a positive correlation between *B. cereus* abundance and deltamethrin resistance in field populations and confirmed that reintroduction of the bacterium into antibiotic-treated mosquitoes restored resistance levels.

Our mechanistic investigations revealed that *B. cereus*_HL4.2 reduces deltamethrin susceptibility through two distinct but complementary pathways. First, infection enhances the activity of key detoxification enzymes, particularly P450s and GSTs, which are known to play crucial roles in pyrethroid metabolism [53–58]. A previous study of *Ae. albopictus* found that mosquitoes oral feeding with *Serratia oryzae* significantly increased both the mosquito resistance to deltamethrin and their metabolic detoxification enzyme activities including P450, GST, and esterase [30]. This enhanced detoxification capacity likely enables mosquitoes to metabolize and eliminate the insecticide more efficiently, but the detailed mechanism requires further investigation. Second, our in vitro experiments demonstrated that *B. cereus*_HL4.2 can directly utilize deltamethrin as a carbon source, suggesting that the bacterium can degrade the insecticide within the mosquito gut, thereby reducing its effective concentration and toxicity. However, the mosquito gut represents a complex environment involving multiple microbial interactions, host factors, and diverse physiological conditions that may influence degradation efficiency. Moreover, although we measured detoxification enzyme activities and in vitro degradation, other pathways potentially contributing to deltamethrin resistance were not examined in this study. While *B. cereus* infection, elevated detoxification enzyme activities, and altered insecticide susceptibility occurred simultaneously, a direct causal relationship between bacterial infection and enzyme upregulation requires further investigation.

It is important to recognize that the heterogeneity of mosquito habitats may lead to the presence of distinct microorganisms that promote insecticide resistance in different geographic areas. Nevertheless, the underlying mechanisms through which such bacteria enhance the detoxification enzyme activity of their mosquito hosts remain to be elucidated. In the study examining *B. cereus*_HL4.2 infection and the restoration of deltamethrin resistance in antibiotic-treated *Ae. albopictus*, it should be noted that antibiotic treatment itself may exert subclinical toxic effects on mosquitoes. In addition, alterations in the overall gut microbiota composition resulting from antibiotic exposure and subsequent mono-association with *B. cereus* could also influence resistance outcomes. The resistance mechanisms—bacterial degradation of the insecticide and the induction of host detoxification enzymes—jointly provide mosquitoes with a robust defense against deltamethrin [54, 55]. This multi-layered protection may explain the rapid development of resistance in field populations where *B. cereus* is prevalent. Similar mechanisms have been observed in other insect–microbe systems, such as the gut bacterium D39 in *Delia antiqua*, which degrades phoxim and increases host resistance [59], and *Serratia oryzae* in *Ae. albopictus* [30], which enhances both deltamethrin resistance and detoxification enzyme activities. It is plausible that the two proposed mechanisms—direct bacterial degradation of the insecticide and the induction of host detoxification pathways—are not mutually exclusive but may function synergistically. To unequivocally delineate the contribution of each mechanism, future work should employ targeted approaches, such as the use of specific P450/GST inhibitors or RNA interference (RNAi) to knock down key detoxification genes, in the presence or absence of the symbiont.

It is common that mosquitoes increase their insecticide resistance by altering their gene expression [14], evidenced by the report that the high-expression of *CYP6P3* and *CYP6M2* have been shown to increase pyrethroid resistance in mosquitoes [54]. The high expression of these genes is due to the chronic exposure of mosquitoes to insecticide pressure. Transcriptome analysis in our study revealed that *B. cereus*_HL4.2 infection alters the expression of genes involved in multiple metabolic and signaling pathways. This study also revealed an upregulation of genes related to the cAMP signaling system. It has been shown that silencing GPCR-related genes in resistant *Culex quinquefasciatus* reduces resistance to permethrin and downregulates P450 detoxification enzyme expression, indicating that the GPCR signaling pathway may participate indirectly in the metabolism of pyrethroid insecticides through the regulation of detoxification enzyme expression [54, 60]. Taken together, these findings suggest that the GPCR–cAMP signaling system plays important regulatory roles in the development and maintenance of insecticide resistance in mosquitoes. Interestingly, the downregulation of genes associated with ABC transporters was unexpected, as these proteins typically contribute to insecticide resistance by facilitating toxin efflux [61, 62]. We hypothesized that *B. cereus*_HL4.2 may facilitate the metabolism of toxic substances, such as insecticides, to such an extent that efflux mechanisms become less critical. However, further research is needed to fully elucidate the functional significance of these transcriptomic changes.

In our GFP-tracking experiments, we also modeled the accumulation in the gut process of *B. cereus* during the development of *Ae. albopictus* after acquiring *B. cereus*_HL4.2 and found that *B. cereus*_HL4.2 could not vertically transmit to the next generation after oral infection. This suggests that mosquitoes acquire these beneficial bacteria from their larval habitats, particularly environments contaminated with organic compounds and insecticides [63–65]. Indeed, *B. cereus* is reportedly widespread in the environment and in the insects’ gut [63]. *Bacillus cereus* has been reported to degrade not only pyrethroids but also water environmental pollutants such as phenol, which supports our speculation [65]. Furthermore, Huang et al. isolated a strain of the heavy-metal-tolerant bacterium *B. cereus* BCS1 in polluted soil that degraded pyrethroids [64]. These polluted soil and water environments with organic compounds promote the growth of *B. cereus*. It is likely that mosquito larval stage acquires these bacteria from these polluted environments [66], and thus are naturally tolerant to insecticides.

## Conclusions

This multifaceted investigation establishes a mechanistic link between mosquito gut symbiotic bacterium *B. cereus*_HL4.2 and reduced deltamethrin susceptibility in *Ae. albopictus*, operating through integrated metabolic and enzymatic degradation pathways. These results highlight the importance of considering host–microbiome interactions in understanding and managing insecticide resistance, potentially opening new avenues for mitigating this growing challenge to vector control and disease prevention.

## Supplementary Information

Additional file 1. Table S1 Geographic coordinates and collection details for field *Aedes albopictus* populations from five locations in Hainan Province, China.

Additional file 2. Table S2 Primer list for PCR, qPCR, and RT-qPCR analyses in this study.

Additional file 3. Table S3 RNA-seq and RT-qPCR validation results of laboratory-susceptible *Ae. albopictus* before and after *B. cereus* infection.

Additional file 4. Table S4. KEGG pathway enrichment analysis of differentially expressed genes in laboratory-susceptible *Ae. albopictus* before and after infection with *B. cereus*.

Additional file 5. Table S5. GO enrichment analysis of differentially expressed genes in laboratory-susceptible *Ae. albopictus* before and after infection with *B. cereus*.

Additional file 6. Fig S1 Morphological characterization of* B. cereus*_HL4.2. (a) Colony morphology of *B. cereus* on Brain-Heart Infusion Broth (BHI) plates showing typical white, flat colonies with irregular edges. (b) Gram stain of *B. cereus* demonstrating characteristic Gram-positive rod-shaped bacteria*.*

Additional file 7. Fig S2 Mosquito gut samples cultured before and after antibiotic treatment in LB medium. (a) Control group with sterile PBS. (b) Gut homogenate of mosquitoes before antibiotic treatment (n=20). (c) Gut homogenate of mosquitoes after antibiotic treatment (n=20).

Additional file 8. Fig S3 Degradation of different concentrations of deltamethrin by *B. cereus*_HL4.2. (a) dose-dependent growth of *B. cereus* on inorganic salt plates supplemented with 0 mg/L, 50 mg/L, 100 mg/L, 200 mg/L of deltamethrin. (b) Quantification of *B. cereus* colony-forming units (CFU) on inorganic salt plates with 0 mg/L, 50 mg/L, 100 mg/L, and 200 mg/L deltamethrin.

## Data Availability

The sequencing data have been deposited in the Short Read Archive (NCBI) with a BioProject accession number (PRJNA1237220).
