# Supplementary figures and images for: Aedes albopictus gut symbiotic bacterium Bacillus cereus improves its deltamethrin resistance
Source: Parasit Vectors. 2026 Jan 9;19:72. doi: 10.1186/s13071-025-07229-5 (PMC12882415; doi:10.1186/s13071-025-07229-5)

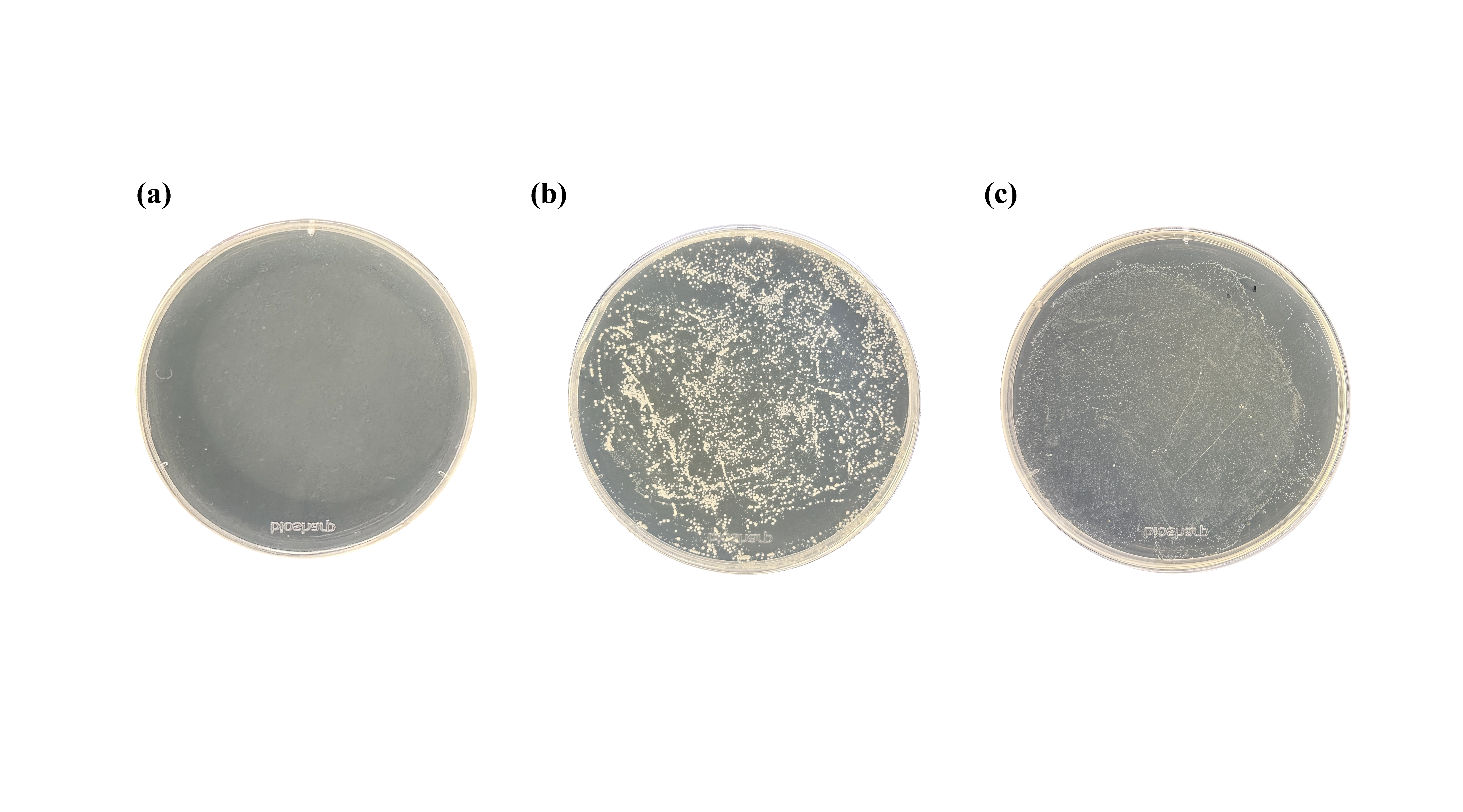

Supplement: Supplementary file 2 — Additional file 2. Table S2 Primer list for PCR, qPCR, and RT-qPCR analyses in this study. [file 13071_2025_7229_MOESM2_ESM.tif]
