# Supplementary material for: Aedes albopictus gut symbiotic bacterium Bacillus cereus improves its deltamethrin resistance
Source: Parasit Vectors. 2026 Jan 9;19:72. doi: 10.1186/s13071-025-07229-5 (PMC12882415; doi:10.1186/s13071-025-07229-5)
Supplement: Supplementary file 4 — Additional file 4. Table S4. KEGG pathway enrichment analysis of differentially expressed genes in laboratory-susceptible Ae. albopictus before and after infection with B. cereus. [file 13071_2025_7229_MOESM4_ESM.docx]

| Abbreviation | City or County | Coordinates | Date |
| --- | --- | --- | --- |
| HK | Haikou | 19°59′01″ N, 110°19′54″ E | 2022.06.13 |
| WC | Wenchang | 19°33′07″ N, 110°47′29″ E | 2023.07.12 |
| DZ | Danzhou | 19°32′22″ N, 109°13′27″ E | 2023.10.01 |
| LD | Ledong | 18°45′03″ N, 109°10′19″ E | 2023.09.03 |
| DA | Dingan | 19°41′41″ N, 110°21′51″ E | 2023.06.10 |

**Table S1.** Information on field *Aedes albopictus* collection in Hainan Province
