# Supplementary material for: Aedes albopictus gut symbiotic bacterium Bacillus cereus improves its deltamethrin resistance
Source: Parasit Vectors. 2026 Jan 9;19:72. doi: 10.1186/s13071-025-07229-5 (PMC12882415; doi:10.1186/s13071-025-07229-5)
Supplement: Supplementary file 5 — Additional file 5. Table S5. GO enrichment analysis of differentially expressed genes in laboratory-susceptible Ae. albopictus before and after infection with B. cereus. [file 13071_2025_7229_MOESM5_ESM.docx]

**Table S2**. Primers for amplification

| Target gene name or ID | Primer name | Sequence (5'-3') |
| --- | --- | --- |
| *gyrB* | *gyrB*-F | GCCCTGGTATGTATATTGGATCTAC |
|  | *gyrB*-R | GGTCATAATAACTTCTACAGCAGGA |
| LOC109417371 | F | CTCGACGACTCCCGAATTCAT |
|  | R | GTCGCAGCTGTAGGGATCTGG |
| LOC115262585 | F | AAACCTGAACAATGTGAAAG |
|  | R | GTTCATGATCACACACTCG |
| LOC115254208 | F | TTCATAACCCCGTACCTGCTG |
|  | R | TTGCGTAGCGTTGATTGGAC |
| LOC109410574 | F | GCACCGTTTCAAGCCTTCTG |
|  | R | TCAATCAGCAGCTCGTG |
| LOC109397048 | F | GACCGAGTTTGGCAGTTGTAT |
|  | R | TGCATTGCTCTTCGCCGTCGT |
| LOC115258427 | F | CTTTGCTCTGCTTCTCGCTG |
|  | R | AGCTCCGATCCAATTCAACTCC |
| LOC109428377 | F | AGTATCGAGCAGACCCCAAC |
|  | R | GTCTATTTTGGCGGGCGTTT |
| *β-actin* | F | GCCGTCTTCCCGTCCAT |
|  | R | GGCGACACGCAGCTCATT |

*Note*: *β-actin* was selected and used as the reference gene.

*Abbreviation*: F, forward primer; R, reverse primer.
