# Supplementary material for: Aedes albopictus gut symbiotic bacterium Bacillus cereus improves its deltamethrin resistance
Source: Parasit Vectors. 2026 Jan 9;19:72. doi: 10.1186/s13071-025-07229-5 (PMC12882415; doi:10.1186/s13071-025-07229-5)
Supplement: Supplementary file 6 — Additional file 6. Fig S1 Morphological characterization of B. cereus_HL4.2. (a) Colony morphology of B. cereus on Brain-Heart Infusion Broth (BHI) plates showing typical white, flat colonies with irregular edges. (b) Gram stain of B. cereus demonstrating characteristic Gram-positive rod-shaped bacteria. [file 13071_2025_7229_MOESM6_ESM.docx]

**Table S3.** RNA-seq and RT-qPCR validation results of *Ae. albopictus* before and after *B. cereus* infection.

| **gene ID** | **gene name** | **log**2**FC** | **2^**ΔΔCt |
| --- | --- | --- | --- |
| 109406068 | unconventional prefoldin RPB5 interactor-like protein | 22.85 | — |
| 109409029 | 27 kDa hemolymph protein-like | 22.30 | — |
| 115267760 | uncharacterized LOC115267760 | 5.88 | — |
| 115268788 | uncharacterized LOC115268788 | 5.04 | — |
| 115266071 | uncharacterized LOC115266071 | 4.54 | — |
| 109398597 | uncharacterized LOC109398597 | 2.21 | — |
| 109417371 | uncharacterized LOC109417371 | 2.18 | 3.82 |
| 109431230 | histone H1 | 2.17 | — |
| 115263536 | uncharacterized LOC115263536 | 2.17 | — |
| 115260919 | uncharacterized LOC115260919 | 1.74 | — |
| 115264623 | histone H1-like | 1.47 | — |
| 109421135 | angiopoietin-4-like | 1.42 | — |
| 115264617 | histone H3 | 1.35 | — |
| 115262585 | uncharacterized LOC115262585 | 1.13 | 3.82 |
| 115254208 | angiopoietin-4-like | 1.11 | 3.39 |
| 109410574 | ficolin-3-like | 1.03 | 2.87 |
| 109410594 | angiopoietin-4 | 1.03 | — |
| 109398787 | putative nuclease HARBI1 | 0.99 | — |
| 109405244 | uncharacterized LOC109405244 | 0.89 | — |
| 109431292 | probable helicase with zinc finger domain | 0.74 | — |
| 109424349 | transcription factor Ouib-like | 0.68 | — |
| 109427254 | THO complex subunit 7 homolog | 0.66 | — |
| 115256483 | zinc finger protein 2-like | 0.63 | — |
| 115254309 | zinc finger protein with KRAB and SCAN domains 1-like | 0.57 | — |
| 115258846 | uncharacterized LOC115258846 | 0.50 | — |
| 109414810 | adenylyl cyclase 78C | 0.45 | — |
| 109408290 | prolyl 3-hydroxylase sudestada1 | 0.40 | — |
| 109400199 | interleukin enhancer-binding factor 2 homolog | 0.39 | — |
| 109432909 | putative defense protein Hdd11-like | 0.38 | — |
| 109414230 | augmin complex subunit dgt5 | 0.34 | — |
| 109399400 | DE-cadherin | 0.34 | — |
| 109398460 | 4-hydroxybutyrate coenzyme A transferase | -0.35 | — |
| 109411467 | death-associated inhibitor of apoptosis 1 | -0.38 | — |
| 109408659 | xanthine dehydrogenase 1-like | -0.4 | — |
| 115268591 | lysosomal alpha-mannosidase-like | -0.4 | — |
| 109422947 | ATP-binding cassette sub-family D member 3 | -0.43 | — |
| 109427110 | proteasome subunit alpha type-2 | -0.44 | — |
| 109418259 | probable ribonuclease ZC3H12B | -0.47 | — |
| 109412112 | glycolipid transfer protein-like | -0.47 | — |
| 109399594 | DNA-binding protein D-ETS-4 | -0.5 | — |
| 115253594 | uncharacterized LOC115253594 | -0.51 | — |
| 109430960 | protein artichoke-like | -0.52 | — |
| 109427990 | galectin-12 | -0.52 | — |
| 109409041 | 27 kDa hemolymph protein-like | -0.53 | — |
| 115265356 | uncharacterized LOC115265356 | -0.54 | — |
| 109428453 | probable phospholipid-transporting ATPase IF | -0.54 | — |
| 109400483 | uncharacterized LOC109400483 | -0.57 | — |
| 109400094 | multidrug resistance-associated protein 9-like | -0.59 | — |
| 115260920 | serine protease inhibitor 28Dc-like | -0.61 | — |
| 109411696 | lipoma-preferred partner homolog | -0.61 | — |
| 109417818 | ATP-binding cassette sub-family A member 3-like | -0.62 | — |
| 109406624 | uncharacterized LOC109406624 | -0.62 | — |
| 115269307 | sodium/potassium-transporting ATPase subunit alpha-like | -0.63 | — |
| 109427893 | titin | -0.64 | — |
| 109422416 | uncharacterized LOC109422416 | -0.64 | — |
| 115259581 | annexin B10-like | -0.65 | — |
| 115260685 | uncharacterized LOC115260685 | -0.67 | — |
| 109425177 | ankyrin repeat and SAM domain-containing protein 4B-like | -0.68 | — |
| 115260002 | uncharacterized LOC115260002 | -0.69 | — |
| 109429051 | lysozyme c-1-like | -0.69 | — |
| 109413806 | uncharacterized LOC109413806 | -0.69 | — |
| 109417169 | uncharacterized LOC109417169 | -0.69 | — |
| 109402345 | gamma-butyrobetaine dioxygenase | -0.71 | — |
| 109408662 | xanthine dehydrogenase/oxidase-like | -0.71 | — |
| 109422969 | lysophosphatidylcholine acyltransferase | -0.71 | — |
| 109430044 | dual specificity protein phosphatase 10-like | -0.74 | — |
| 109427739 | peroxidase | -0.75 | — |
| 109428378 | pancreatic triacylglycerol lipase-like | -0.75 | — |
| 115268057 | uncharacterized LOC115268057 | -0.76 | — |
| 109408413 | leucine-rich repeat-containing G-protein coupled receptor 4-like | -0.77 | — |
| 115253465 | alpha-amylase A-like | -0.77 | — |
| 109416369 | tyrosine-protein phosphatase vhp-1 | -0.79 | — |
| 115255219 | venom allergen 5-like | -0.79 | — |
| 109404624 | sialin-like | -0.8 | — |
| 109430163 | annulin-like | -0.81 | — |
| 109419754 | protein Tob2 | -0.81 | — |
| 109421559 | probable cytochrome P450 6a14 | -0.82 | — |
| 109407536 | uncharacterized LOC109407536 | -0.82 | — |
| 109426935 | sodium-dependent nutrient amino acid transporter 1-like | -0.82 | — |
| 109409872 | angiopoietin-related protein 6-like | -0.83 | — |
| 109398755 | cyclin-dependent kinase 7 | -0.84 | — |
| 115270450 | integrin alpha-PS3-like | -0.84 | — |
| 115270101 | leucine-rich repeat-containing G-protein coupled receptor 4-like | -0.88 | — |
| 109420938 | 2-oxo-4-hydroxy-4-carboxy-5-ureidoimidazoline decarboxylase-like | -0.88 | — |
| 109401330 | protein phosphatase 1 regulatory inhibitor subunit PPP1R7 homolog | -0.9 | — |
| 109424440 | calpain-B-like | -0.91 | — |
| 109429981 | ATP-binding cassette sub-family G member 4 | -0.95 | — |
| 109429983 | stress-activated protein kinase JNK-like | -0.95 | — |
| 109403398 | phenoloxidase-activating factor 2 | -0.95 | — |
| 109428418 | uncharacterized LOC109428418 | -0.98 | — |
| 109410283 | UDP-glucuronosyltransferase 2B18-like | -0.98 | — |
| 115268314 | uncharacterized LOC115268314 | -0.98 | — |
| 115267832 | farnesol dehydrogenase-like | -0.99 | — |
| 109400485 | prolyl 3-hydroxylase sudestada1-like | -1.02 | — |
| 109430119 | uncharacterized LOC109430119 | -1.02 | — |
| 109397048 | uncharacterized LOC109397048 | -1.04 | 0.43 |
| 109409856 | myosin-M heavy chain-like | -1.06 | — |
| 109410676 | C-type lectin 37Db-like | -1.09 | — |
| 109423630 | zinc metalloproteinase-disintegrin-like BmMP | -1.1 | — |
| 109429642 | clavesin-1-like | -1.11 | — |
| 115269405 | farnesol dehydrogenase-like | -1.11 | — |
| 115258427 | perlucin-like | -1.13 | 0.13 |
| 109411693 | sialin | -1.14 | — |
| 109417369 | uncharacterized LOC109417369 | -1.15 | — |
| 115253896 | tetraspanin-2A-like | -1.17 | — |
| 115266493 | ficolin-2-like | -1.17 | — |
| 115259905 | mite group 2 allergen Lep d 2-like | -1.19 | — |
| 115264125 | disintegrin and metalloproteinase domain-containing protein 12-like | -1.19 | — |
| 109428377 | pancreatic lipase-related protein 2-like | -1.22 | 0.35 |
| 109400781 | farnesol dehydrogenase-like | -1.23 | — |
| 109433005 | uncharacterized LOC109433005 | -1.27 | — |
| 109426656 | L-threonine ammonia-lyase-like | -1.31 | 0.67 |
| 109399573 | farnesol dehydrogenase-like | -1.44 | — |
| 109412674 | toll-like receptor 8 | -1.48 | — |
| 109400899 | uncharacterized LOC109400899 | -1.61 | — |
| 115259650 | mediator of RNA polymerase II transcription subunit 27 | -1.86 | — |
| 115260572 | uncharacterized LOC115260572 | -2.91 | — |
| 109419895 | uncharacterized LOC109419895 | -3.26 | — |
| 115267204 | inositol oxygenase-like | -3.32 | — |
| 109432834 | putative fatty acyl-CoA reductase CG5065 | -3.65 | — |
| 109417423 | uncharacterized LOC109417423 | -6.59 | — |
| 109432536 | protein nessun dorma-like | -21.64 | — |

**Note:** Log_2_Fold Change > 0 indicates upregulated genes, and log_2_Fold Change < 0 indicates downregulated genes. The control group represents samples collected prior to infection with *B. cereus*, while the treatment group represents samples collected following infection. 2^^ΔΔCt^ refers to the differential expression multiple.
