# Supplementary material for: Aedes albopictus gut symbiotic bacterium Bacillus cereus improves its deltamethrin resistance
Source: Parasit Vectors. 2026 Jan 9;19:72. doi: 10.1186/s13071-025-07229-5 (PMC12882415; doi:10.1186/s13071-025-07229-5)
Supplement: Supplementary file 7 — Additional file 7. Fig S2 Mosquito gut samples cultured before and after antibiotic treatment in LB medium. (a) Control group with sterile PBS. (b) Gut homogenate of mosquitoes before antibiotic treatment (n=20). (c) Gut homogenate of mosquitoes after antibiotic treatment (n=20). [file 13071_2025_7229_MOESM7_ESM.docx]

Table S4. KEGG pathway enrichment analysis of differentially expressed genes in *Ae. albopictus* before and after infection with *B. cereus*.

| KEGG Pathway Term Description | KEGG Pathway Term ID | KEGG Pathway Term Level2 | Term Candidate Gene Number | Term Gene Number | Rich Ratio | P value |
| --- | --- | --- | --- | --- | --- | --- |
| Drug metabolism - other enzymes | 983 | Xenobiotics biodegradation and metabolism | 3 | 138 | 0.022 | 0.049 |
| Ascorbate and aldarate metabolism | 53 | Carbohydrate metabolism | 2 | 58 | 0.034 | 0.047 |
| Endocrine resistance | 1522 | Drug resistance: antineoplastic | 2 | 56 | 0.036 | 0.044 |
| Inflammatory mediator regulation of TRP channels | 4750 | Sensory system | 2 | 56 | 0.036 | 0.044 |
| **Gastric acid secretion** | **4971** | **Digestive system** | **2** | **53** | **0.038** | **0.040** |
| **Thyroid hormone synthesis** | **4918** | **Endocrine system** | **2** | **52** | **0.038** | **0.038** |
| Valine, leucine and isoleucine biosynthesis | 290 | Amino acid metabolism | 1 | 5 | 0.200 | 0.030 |
| Apoptosis - multiple species | 4215 | Cell growth and death | 2 | 42 | 0.048 | 0.026 |
| **Toll and Imd signaling pathway** | **4624** | **Immune system** | **3** | **101** | **0.030** | **0.022** |
| Carbohydrate digestion and absorption | 4973 | Digestive system | 2 | 35 | 0.057 | 0.018 |
| Apoptosis - fly | 4214 | Cell growth and death | 3 | 93 | 0.032 | 0.018 |
| **Bile secretion** | **4976** | **Digestive system** | **3** | **92** | **0.033** | **0.017** |
| Peroxisome | 4146 | Transport and catabolism | 4 | 165 | 0.024 | 0.016 |
| **Salivary secretion** | **4970** | **Digestive system** | **3** | **86** | **0.035** | **0.014** |
| **Purine metabolism** | **230** | **Nucleotide metabolism** | **4** | **152** | **0.026** | **0.012** |
| Caffeine metabolism | 232 | Biosynthesis of other secondary metabolites | 2 | 19 | 0.105 | 0.006 |
| ABC transporters | 2010 | Membrane transport | 4 | 67 | 0.060 | 0.001 |
| Insect hormone biosynthesis | 981 | Metabolism of terpenoids and polyketides | 4 | 66 | 0.061 | 0.001 |
| Terpenoid backbone biosynthesis | 900 | Metabolism of terpenoids and polyketides | 4 | 64 | 0.063 | 0.001 |

**Note:** The P-values indicate the statistical significance of these enrichment results, with lower values representing more robust findings. The Rich Ratio represents the proportion of genes from the input list involved in the specific KEGG Pathway relative to the total number of genes annotated with that term. The highlighted text represents the enriched up-regulated pathways, with the remainder indicating down-regulated pathways.
