# Supplementary material for: Aedes albopictus gut symbiotic bacterium Bacillus cereus improves its deltamethrin resistance
Source: Parasit Vectors. 2026 Jan 9;19:72. doi: 10.1186/s13071-025-07229-5 (PMC12882415; doi:10.1186/s13071-025-07229-5)
Supplement: Supplementary file 8 — Additional file 8. Fig S3 Degradation of different concentrations of deltamethrin by B. cereus_HL4.2. (a) dose-dependent growth of B. cereus on inorganic salt plates supplemented with 0 mg/L, 50 mg/L, 100 mg/L, 200 mg/L of deltamethrin. (b) Quantification of B. cereus colony-forming units (CFU) on inorganic salt plates with 0 mg/L, 50 mg/L, 100 mg/L, and 200 mg/L deltamethrin. [file 13071_2025_7229_MOESM8_ESM.docx]

**Table S5. GO enrichment analysis of differentially expressed genes in *Ae. albopictus* before and after infection with *B. cereus*.**

| GO Enrichment Functional Classification | GO Term ID | Gene Description | Gene Number | Rich Ratio | P-value |
| --- | --- | --- | --- | --- | --- |
| Molecular Function | GO:0003986 | acetyl-CoA hydrolase activity | 1 | 1.000 | 0.005 |
|  | GO:0008138 | protein tyrosine/serine/threonine phosphatase activity | 1 | 1.000 | 0.005 |
|  | GO:0008775 | acetate CoA-transferase activity | 1 | 1.000 | 0.005 |
|  | **GO:0004016** | **adenylate cyclase activity** | **2** | **0.500** | **0.011** |
|  | GO:0004725 | protein tyrosine phosphatase activity | 2 | 0.500 | 0.011 |
|  | GO:0005085 | guanyl-nucleotide exchange factor activity | 4 | 0.250 | 0.022 |
|  | GO:0008233 | peptidase activity | 9 | 0.111 | 0.048 |
| Biological Process | GO:0006083 | acetate metabolic process | 1 | 1.000 | 0.004 |
|  | GO:0019679 | propionate metabolic process, methylcitrate cycle | 1 | 1.000 | 0.004 |
|  | **GO:0006171** | **cAMP biosynthetic process** | **2** | **0.500** | **0.008** |
|  | **GO:0035556** | **intracellular signal transduction** | **4** | **0.250** | **0.016** |
|  | **GO:0006334** | **nucleosome assembly** | **12** | **0.083** | **0.047** |

**Note:** The P-values indicate the statistical significance of these enrichment results, with lower values representing more robust findings. The Rich Ratio represents the proportion of genes from the input list involved in the specific GO term relative to the total number of genes annotated with that term. The highlighted text represents the enriched up-regulated pathways, with the remainder indicating down-regulated pathways.
